# “I stretch them out as long as possible:” U.S. women’s experiences of menstrual product insecurity during the COVID-19 pandemic

**DOI:** 10.1186/s12905-023-02333-z

**Published:** 2023-04-15

**Authors:** Margaret L. Schmitt, Katie Dimond, Andrew R. Maroko, Penelope A. Phillips-Howard, Caitlin Gruer, Amanda Berry, Denis Nash, Shivani Kochhar, Marni Sommer

**Affiliations:** 1 Mailman School of Public Health, Columbia University, 722 W 168Th St, New York, NY 10032 USA; 2 Institute for Implementation Science in Population Health, City University of New York (CUNY), New York City, NY USA; 3 Liverpool School of Tropical Medicine, Liverpool, UK

**Keywords:** Menstruation, Period poverty, Women’s health, Gender inequality, COVID-19

## Abstract

**Background:**

A growing body of evidence highlights how the COVID-19 pandemic has exacerbated gender inequalities in the US. This resulted in women being more vulnerable to economic insecurity and decreases in their overall well-being. One relevant issue that has been less explored is that of women’s menstrual health experiences, including how inconsistent access to menstrual products may negatively impact their daily lives.

**Methods:**

This qualitative study, conducted from March through May 2021, utilized in-depth interviews that were nested within a national prospective cohort study. The interviews (*n* = 25) were conducted with a sub-sample of cis-gender women living across the US who had reported challenges accessing products during the first year of the pandemic. The interviews sought to understand the barriers that contributed to experiencing menstrual product insecurity, and related coping mechanisms. Malterud’s ‘systematic text condensation’, an inductive thematic analysis method, was utilized to analyze the qualitative transcripts.

**Results:**

Respondents came from 17 different states across the U.S. Three key themes were identified: financial and physical barriers existed to consistent menstrual product access; a range of coping strategies in response to menstrual product insecurity, including dependence on makeshift and poorer quality materials; and heightened experiences of menstrual-related anxiety and shame, especially regarding the disclosure of their menstruating status to others as a result of inadequate menstrual leak protection.

**Conclusions:**

Addressing menstrual product insecurity is a critical step for ensuring that all people who menstruate can attain their most basic menstrual health needs. Key recommendations for mitigating the impact of menstrual product insecurity require national and state-level policy reform, such as the inclusion of menstrual products in existing safety net basic needs programs, and the reframing of menstrual products as essential items. Improved education and advocacy are needed to combat menstrual stigma.

## Background

There is growing evidence around the world highlighting how the COVID-19 pandemic has exacerbated gender inequalities [1, 2]. Women have been more likely to experience socio-economic hardship throughout the pandemic including higher unemployment and increased caretaking responsibilities; the latter a consequence of both gender norms and the shutdown of schools and childcare facilities [3, 4]. This has resulted in women being more vulnerable to economic insecurity and decreases in their overall well-being [5]. One less understood facet of women’s well-being since the start of the pandemic is that of their “menstrual health.” Menstrual health can be defined as “a state of complete physical, mental, and social well-being… in relation to the menstrual cycle [6].” Sufficient access to an individuals’ preferred menstrual product is considered a fundamental component of achieving menstrual health, along with adequate menstrual health information, sanitation and disposal facilities, and appropriate and timely health services and resources. Menstrual products are broadly defined as any materials used to catch menstrual blood, such as reusable and disposable pads, menstrual cups, and tampons. The conditions created by the pandemic, including socio-economic adversity, supply chain disruptions, and limited social and physical mobility have likely hindered women’s menstrual health in several ways. In this article, we use the terms “woman” and “girl” to refer to cisgender females who experience the biological process of menstruation, while acknowledging that not all women menstruate, and that menstruation is experienced by a broader expanse of gender identities.

In the United States of America (U.S.) there is growing attention to “period poverty” or the inability to afford menstrual products as needed [7]. The limited pre-pandemic evidence on this issue in the U.S. primarily focused on low-income women and students. A 2019 study of 183 low-income women in St. Louis, Missouri found that 64% of respondents were unable to afford menstrual products in the previous year, with a third of those women disclosing also having to rely on a makeshift menstrual material, such as toilet paper or rags [8]. The same researchers examined menstrual product insecurity amongst low-income St. Louis High School students, finding that of the 119 respondents, 64% had experienced menstrual product insecurity and almost 67% had relied on free products available through school [9]. Such findings are important in illustrating not only the prevalence of period poverty amongst low-income populations, but also the importance of social support systems, such as schools, to help address this unmet need.

﻿Managing menstrual periods can be a stigmatizing experience for girls and women of all ages, especially among low-income populations [8, 10, 11]. ﻿The absence of adequate menstrual health support can lead adolescent girls and women to perceive periods and their management negatively. Such notions are often reinforced by the societal promotion of “menstrual etiquette” norms, which are behavioral expectations related to “acceptable” period management practices (e.g., not revealing one’s menstruating state to others) [12]. These societal notions are often fortified by families, peer groups, and the mass media [13–15]. Such expectations may originate from ongoing menstrual stigma, including that periods are “dirty” and shameful [16, 17]. These menstrual social norms can negatively impact one’s confidence, mental health, and willingness to look for social support when addressing menstrual health issues (Hennegan et al., 2019; Phillips-Howard et al., 2016). Menstrual stigma may have even more significant implications for transgender and non-binary people who menstruate, given the heightened potential for gender dysphoria and anxiety about menstrual leaks being seen by others [18, 19].

Menstrual product insecurity in the U.S. has increased since the start of the pandemic in March 2020; a topic increasingly covered by the news media [20–23]. However, there remain limited U.S. governmental supports or safety nets addressing this issue. Most U.S. public assistance programs, such as the Supplemental Nutrition Assistance Program (SNAP) or the Special Supplemental Nutrition Program for Women, Infants, and Children (WIC) do not allow for the purchase of menstrual products [21] and are already underfunded programs. Menstruating students who may have relied on schools for accessing menstrual products before the pandemic lost access to such supplies due to school shutdowns and shifts to remote learning [24, 25]. The lack of federal assistance or safety nets for accessing menstrual products means that many people experiencing economic hardship may turn to food banks or other social service organizations to locate supplies, some of which saw up to a 60% increase in demand since the start of the pandemic [26].

﻿This paper examines U.S. women’s experiences of menstrual product insecurity during the first year of the COVID-19 pandemic, including its implications on their period management practices, their daily lives, and their perceived well-being.

## Methods

### Research design

This qualitative study conducted from March through May 2021 included a series of In-depth Interviews (IDIs) conducted with cis-gendered women who menstruate. Participants were drawn from a larger quantitative study (*n* = 1496) examining menstrual product insecurity during the early stages of the COVID pandemic in the U.S. [27] which itself was part of the *Communities, Households and SARS-CoV-2 Epidemiology (CHASING)* COVID study, a national, community-based prospective cohort (*n* = 6,753). *CHASING COVID* is designed to help contribute to the understanding of this pandemic, estimate the incidence of SARS-CoV-2 infection, assess the impact of the pandemic on households across the U.S., and assess the uptake of pandemic mitigation strategies [28]. The study received Institutional Review Board (IRB) approval from the Columbia University Medical Center.

### Sampling and recruitment

Study participants were recruited based on their participation in the *CHASING COVID* study which includes respondents living in all 50 U.S. states, ﻿the District of Columbia, Puerto Rico, and Guam [29]. Participants must have been 18 years or older, identified as female, completed the survey in English, and responded to a menstruation-related survey panel of the *CHASING COVID* study as (1) not currently pregnant and (2) has menstruated since March 2020. To ensure IDIs included women experiencing menstrual product insecurity, participants were further filtered based on baseline income (< 49 k), having experienced pandemic-related income loss since March 2020, and having responded “yes” to at least 2 of the 3 period poverty-related questions (see Table 1, *n* = 78). All eligible women were contacted about participation in the study. To capture experiences from different types of environments, respondents were enrolled across rural (*n* = 9), suburban (*n* = 4), and urban (*n* = 12) geographies based on their ZIP code of residence. A total of 25 women (aged 18–45) participated in the IDIs from 17 U.S. states and from each of the five U.S. geographical regions: East, Southeast, Southwest, Midwest, and West. To capture experiences from different types of environments, respondents were enrolled across rural (*n* = 9), suburban (*n* = 4), and urban (*n* = 12) geographies based on their ZIP code of residence. All participants were recruited using email correspondence through our partners at *BLINDED*. Each participant was provided a $50 Amazon gift card for their time.

**Table 1:** Period Poverty Survey Questions

Specific questions included in the menstrual survey panel
*Answered “YES” to 2 of 3 questions specified:*
* 1. Could not afford sanitary products?*
* 2. Had to use a makeshift material because ran out of a menstrual product?*
* 3. Paying for a sanitary product is more difficult?*

### Qualitative data collection methods

A semi-structured qualitative interview guide was utilized to explore the factors which led to menstrual product access issues, coping mechanisms utilized, experiences with food banks and social support resources, and how a lack of consistent access to menstrual products may impact their daily life.

### Data collection

The data collection team included 2 graduate-level trained research staff (MLS, MPH; MS, MSN, DrPH) from Columbia University Mailman School of Public Health. Both members of the interview team were cisgender females who had conducted previous research on the menstrual health experiences of low-resource populations in the U.S. and globally. Prior to data collection, the team conducted reflexivity exercises, which included examining their own positionality in relation to the power dynamics of the interview process. In addition, they also reviewed their own experiences as individuals that menstruate in addition to their personal COVID realities and circumstances given the variation of pandemic-related experiences across the country. All IDIs were conducted via a teleconferencing platform (FreeConferenceCall.com) to a participant’s personal phone. Interviews were conducted in English and lasted between 30 to 60 min. Prior to starting the interviews, the study purpose and objectives were explained to each participant. All participants were over 18 years of age or older and provided informed verbal consent. All interviews were recorded and transcribed.

### Conceptual framework

We used an adaptation of the socio-ecological model (SEM) to assess the multiple levels of influence shaping cis-women’s experiences of menstrual product insecurity during the pandemic, including how they impact an individuals’ menstruation-related behaviors and experiences [30]. This included an exploration of how societal (fears about the pandemic, income loss) and structural or environmental (stockouts, price gauging, quarantine) factors impacted women’s menstrual management practices, confidence and self-esteem, and the overall menstrual experience in relation to the shifting household and/or external environments secondary to the pandemic. The conceptual framework also guided an examination of how menstrual product insecurity impacted interpersonal interactions and individual level experiences.

### Data analysis

A two-person cisgender female research team, comprised of a researcher (MLS, MPH) and a graduate student (KD, MPH), reviewed, and uploaded all IDI transcripts into Dedoose. The data was then analyzed using Malterud’s systematic text condensation, ‘an explorative and descriptive thematic analysis method’ [31]. This pragmatic analysis method was selected given its ability to enable for intersubjectivity, reflexivity and its overall feasibility while still enabling for a sufficient level of methodological quality [31]. Furthermore, systematic text condensation is a descriptive approach that enables for the presentation of participant experience as expressed by themselves; a methodological attribute valued by the research team when conducting exploratory research on menstrual health experiences. This method involved four key steps, including a) distilling broad impressions (reviewing all data), b) identification of the key themes (refining themes into specific codes), c) condensing the text from the code and exploring meaning (reducing data into select groups of meaningful codes), and d) synthesizing (developing plausible narratives that highlight the original study questions). A combination of a priori and in vivo coding techniques were utilized for the development of the codebook (step b), which included the initial development of a hierarchal codebook based on key themes derived from the literature and then supplementing this with additional codes generated during the coding process. Previous literature utilized to inform the development of the a priori codes included a series of pre-pandemic U.S. period poverty articles highlighting the challenges faced by populations experiencing homelessness [32, 33] and students and low-income women living in St. Louis [8, 9]. Every transcript was double coded by the two research team members. When disagreements with a code choice were identified, a discussion was conducted by the coders to reach consensus. Key themes were then distributed across the larger research team for further validation, discussion, and the generation of consensus.

## Results

Three key thematic areas were identified during the analysis: 1) financial and physical barriers to menstrual product access; 2) the adoption of coping strategies in response to menstrual product insecurity; and 3) heightened experiences of menstrual-related anxiety and shame.

### Financial and physical barriers to menstrual product access

Almost all the respondents indicated experiencing some financial challenges in purchasing menstrual products since the start of the COVID-19 pandemic. These new challenges were the result of increased financial insecurity within their households, which were attributed to employment status shifts such as reduced hours, lay-offs, or needing to resign due to increased childcare responsibilities. Changes in financial situations, including overall income loss, led many participants to shift from their preferred menstrual product brand or type to generic or lower-cost products, or in some cases to makeshift menstrual materials such as toilet paper or paper towels.

In addition to economic-related barriers, numerous respondents indicated a high frequency of menstrual product “stockouts” and a diminished variety of product brands and types at many local stores during the early phases of the pandemic. As one rural North Carolina woman explained, “*a lot of times, on the shelves, the products available were not for me, they were for lighter [menstrual] flows.”* Using an inadequate menstrual product, such as one designed for lighter bleeding, led women with heavier periods to need a greater quantity of products, which was both costlier and reported to be less effective in terms of reducing menstrual leaks.

Numerous respondents indicated that the supplies available at local stores varied widely over time during these early months of the pandemic. One woman living in an urban part of California described her experiences with inconsistent access, explaining how *“it’s been very scarce…it’s like one week there’ll be some and then when I go next month to get it, they will be completely out of stock.”* Other menstruation supportive supplies, such as pain medication for managing menstrual cramps, were also cited as difficult to find at times. This same respondent from California also noted how:

*…the cramping medication, like the Midol and stuff, that was hard too…they weren’t re-stocking certain areas of the store and were more focused on groceries and…like toilet paper, paper towels and disinfectant type of stuff…*

Many women also reported their preferences for buying menstrual products in bulk during this time, both to reduce anxiety about future product shortages and to minimize COVID-19 exposure risks in stores. One rural woman in Florida explained how her discomfort in stores motivated the rationing of supplies: *“we were terrified to even go into a store the first 3 months…we used everything, include pads, sparingly.”* Some respondents also indicated transportation-related challenges for accessing menstrual products in stores, especially those relying on public transportation. Although a primary reason was concern about COVID-19 exposure, a few participants noted how changes in public transportation operations, including reduced schedules and ridership quotas, created further logistical challenges.

Both poor product selection available at stores and feelings of apprehension in physically accessing them led many respondents to explore online retailers for the first time to purchase menstrual products. Despite the perceived convenience of this approach, many women described fluctuating prices for products during the first year of COVID-19, with the prices oftentimes found to be much higher on some online shopping platforms, such as Amazon. As one rural respondent from North Carolina clarified:

*…sometimes I would order them online and I might not get what I needed to get in time…and sometimes the prices would be way higher online, and I wouldn’t have any choices because a lot of things would be sold out. Things I really needed would be sold out…*

Respondents also described other challenges when ordering online, including product stockouts and longer than normal delivery times, especially for those in rural areas. Buying online thus did not always mitigate women’s frustrations with the pandemic-related limitations of in-person shopping. A small number of women also described personal challenges with shifting to online purchasing for period products, including a lack of a credit line or being unable to afford internet and phone data access for periods of time.

The negative consequences of poor access to quality menstrual products were more acute for women who reported having heavy menstrual periods, menstrual disorders (e.g., fibroids, endometriosis), or physical disabilities. A few respondents indicated how heavy menstrual flows required them to use more products for a longer duration than is recommended. This gave them less flexibility around what types of products would be effective to avoid having menstrual leaks. These women often described having identified, over the course of their menstrual lifespan, specific products that were designated and most dependable for heavy periods. However, the shortages found in stores and online meant that these products could be difficult to find at various points during the first pandemic year. A suburban respondent from Texas explained this predicament: *“I have uterine fibroids…so my periods are extremely heavy… so when all you have is a panty liner left…you wanna talk about being a nervous wreck? It was less than pleasant.”* In addition to women experiencing heavy bleeding, the limited product varieties available in stores was also identified as problematic for women with physical disabilities, including those using wheelchairs. These respondents also described having very specific menstrual product needs related to sizing and absorbency, which could be increasingly challenging to locate giving the fluctuation of supplies during the first year of the pandemic.

### The adoption of coping strategies in response to menstrual product insecurity

All respondents indicated modifying their menstruation management behaviors to some extent, with many indicating adopting specific coping practices to address frequent menstrual product shortages. This included wearing products for longer durations than was recommended or was considered comfortable. As one rural woman in California explained, *“we barely could afford them at times, so I’d stretch them out as long as possible…I took more showers throughout the day, it was the only way I could.”* Elongating use of a product for a longer duration was especially challenging given that many respondents had shifted to cheaper, and often lower quality product brands. One participant living in an urban area of Oklahoma described this specific predicament: *“I would try to make them [tampons] last a whole 8 hours, but sometimes it wasn’t possible because I was using the cheaper brands,”* implying that leaking occurred with prolonged use. Numerous respondents across the various regions of the U.S. indicated how such lower quality, cheaper menstrual product brands made women feel vulnerable to leaking. As a rural woman from North Carolina described:

*…sometimes I would have to buy the way cheaper brand of pads that were no good at all and didn’t really work for me and I was always messing up my clothes. And I didn’t feel good at all, and it was uncomfortable…*

Many respondents indicated that local “dollar stores” were some of the most reliable and affordable locations for purchasing menstrual products during the first year of the pandemic, despite the perceived inferiority of their menstrual products. While other local stores and supermarkets were often prone to stockouts, the dollar stores typically had reliable product supplies. As a rural woman from California explained, *“the dollar store always did have the really cheap pads, so I would try and grab quite a few of those and then try and make those last.”* When probed about quality issues with the cheaper menstrual products, respondents indicated challenges with poor product absorbency (e.g., the ability to soak up blood), defective liners (e.g., the sticky layer which attaches a pad to underwear), comfort, and the limited selection (e.g., different absorbency levels or sizes). One urban woman from Texas explained her own issues with depending on “dollar store” menstrual products:

*…the dollar store products don’t really have the plastic [tampon applicators] or the comfort, they sell cardboard [tampon applicators] and everything is pretty much uncomfortable…and pads, they’re bulkier and they don’t stick very well [to underwear].”*

Challenges with accessing disposable menstrual pads and tampons led a few respondents to try reusable menstrual products for the first time. Reusable products are a segment of period products designed to be used multiple times (with proper cleaning in between uses), and include products such as period underwear, cloth pads, and menstrual cups. One suburban respondent in Maryland indicated trying a menstrual cup for the first time upon realizing the potential cost benefits:

*…I bought a menstrual cup because I figured that… I think the one that I bought cost $25 [U.S.] dollars…so I figured that was gonna be more cost efficient and I wouldn’t run into running out of tampons or pads or anything like that...*

Potential longer term cost savings from reusable products led a few other respondents to try out period underwear for the first time, often purchased online or acquired through donations, such as at food banks or churches. However, a few respondents reported encountering challenges when experimenting with period underwear. One urban respondent in California described what happened when she used a low-cost period underwear purchased online for the first time: *“I reverted to getting menstrual underwear and those don’t work! No, they don’t work…it says that it holds up to two tampons worth or three pads worth…but no they don’t!”* There was, however, variance in the acceptability of these reusable products for the women who had tried them, likely based on the specific product type, brand, and material quality. Beyond potential cost benefits, perceived additional protection against menstrual leaks offered by the period underwear was also cited as a motivating factor, especially for those women who experienced heavy bleeding. Period underwear was viewed as a secondary layer of protection for preventing “embarrassing” stains.

Several women also indicated having to create makeshift menstrual products from household items during the first year of the pandemic. This included having to use paper towels, toilet paper, and even rags as makeshift materials for managing their menstrual blood. As one suburban woman in Texas explained: *“I was having to take paper towels and wrap them up and use them for a pad until I could find something…I had to do that more times than I care to remember.”* Toilet paper shortages, a common occurrence for nearly all respondents at some point during 2020, only made this experience more challenging. As one woman in an urban part of New Mexico explained:

*…it’s just something that you just never thought you’d have to do…you want to use toilet paper, but you can’t because there is not enough…so sometimes, I didn’t tell anybody, but sometimes I did use the cloth rag…and I threw it away…it’s not something you really want to share, but it did happen…*

Several women described feelings of embarrassment and shame upon recalling their reliance on makeshift materials for period management in the past year, highlighting some of the potential mental health implications associated with menstrual product insecurity.

### Heightened experiences of menstrual-related anxiety and shame

The inability to consistently access menstrual products coupled with the need to adopt coping mechanisms resulted in many women feeling shame, embarrassment, and anxiety. Nearly all respondents described having experienced some level of worry regarding whether they would be able to find or afford menstrual products each month over the course of the first pandemic year. Some women indicated that this was the first time in their lives they have had to worry about personal shortages of menstrual products and its implications on their daily lives. Many respondents connected these feelings of anxiety to fears of having to disclose their menstruating status to others, such as family or co-workers, due to noticeable blood stains on their clothing or emitting a bad odor. The women expressed frustration around the challenges they faced maintaining discretion while menstruating. This highlights the role of menstrual stigma in the lives of many women, including how it impacts both their home and social lives. Furthermore, an inability to feel “clean” was another complaint shared by many women, an issue compounded by having to use menstrual products for a longer duration than effective or needing to make do with makeshift materials. The participant living in urban New Mexico, elucidated this sentiment:

*…you don’t feel fresh because you’re using a rag and it’s not something that anybody would know but you do, so it makes you act different or just be grouchy…it’s not like you can tell a person you know, ‘I have toilet paper for a pad so excuse me for today,’ you can’t say that…it’s awful that you have to feel that way and not feel clean…*

This led several women to indicate the need to shower or bathe more frequently to combat feelings of “dirtiness,” fears of emitting bad odors, or as a means for managing the blood flow.

Several women explicitly described the negative mental health implications of these experiences, including episodes of embarrassment, low self-esteem, and depression. An urban Texas woman described her mental health struggles: *“I mean any woman is gonna feel depressed about it because the things [menstrual products] aren’t there for them and that’s hard…that’s really hard emotionally, mentally, on any woman.”* Feelings of shame or embarrassment resulted in many women modifying their daily activities. This included the social and physical stressors menstrual product insecurity put on women’s experiences in home, social, and workplace contexts. A few women highlighted the anxiety caused by using poor quality menstrual products while in the workplace. As one woman in an urban part of Colorado explained, *“I was dealing with heaving bleeding and having issues with bleeding through my pants at work…that’s why I got two pairs of the Thinx [reusable period] panties.”* A few women indicated feeling uncomfortable within their own homes when lacking sufficient menstrual protection, with one urban woman from Washington State explaining: “*you’re like self-conscious and you’re just trying to not let other people know*.” A few respondents indicated preferences to not leave their homes at all when forced to manage their periods with poor quality or makeshift products. As a suburban woman in Florida described when using a makeshift material: *“I try and stay home when I don’t have those [disposable] products available.”* Reductions in physical activity were also described by several participants given worries that makeshift or poor-quality products would shift in their underwear and result in leaks. The participant from an urban part of New Mexico described this anxiety when managing her period with makeshift products, which she had to do on several occasions over the course of the first pandemic year:

*…I have six dogs so I have to be really active…even just to come in the backyard and you just don’t know if you’re paper towels are gonna fall out from your leg, and that’s embarrassing, I would die if something like that happened…I shouldn’t have to worry about stuff like that, there’s way more other things I need to focus on…*

Worries about leaks from makeshift and poor-quality products also led several women to describe feeling more self-conscious about their clothing choices. For example, some participants indicated preferences towards wearing tighter clothing, such as leggings or jeans, to help ensure that poorer quality or makeshift menstrual products “stayed in place.” As a suburban Maryland woman explained, *“I was wearing like workout pants, so that way they were tighter…and if I used toilet paper, nothing was gonna fall out.”* Preferences for tighter fitting clothing were conveyed by several women, especially when leaving the home or while sleeping, in addition to preferences for dark or black clothing in anticipation of bloodstains that they wanted to make sure were not obvious to others.

## Discussion

This qualitative study generated important insights into the ways in which menstrual product insecurity impacted the lives of women in the U.S. during the first year of the COVID-19 pandemic. These findings shed light on reasons why women struggled to purchase or even find menstrual products, including how these challenges were more acute for women experiencing heavy bleeding, menstrual disorders, and physical disabilities. Product shortages led many women to shift to using cheaper and oftentimes poorer quality options, resulting in the increased potential for menstrual leaks. The stress associated with being unable to consistently procure menstrual products, coupled with concerns about disclosing their menstrual status to others, led many women to report increased experiences of menstrual-related anxiety and shame. This discomfort was exacerbated by menstrual stigma, which often reinforced the need for women to keep this issue private, including within their homes and workplaces.

Of the various ways in which the pandemic exacerbated menstrual product insecurity, findings from this study suggest that menstrual products are overdue to be treated as an ‘essential item’ in times of economic crisis. There is a small and growing body of evidence being generated in the U.S. and the United Kingdom (U.K.) highlighting estimates of menstrual product insecurity during COVID-19 [34–36]. A survey of 240 people in the U.K. who have experienced ‘period poverty’ or needed help with managing their periods during the pandemic found that 85% experienced difficulties accessing period products, with 30% of that group indicating being unable to afford products and 45% percent unable to visit stores to purchase products when needed. A 2021 survey conducted by Thinx & PERIOD. in the U.S. found that of the 1,010 student respondents, 23% of them indicated having to choose between buying period products and food or clothing. Notably, the study also found that students of color were more adversely impacted by menstrual product insecurity, with nearly half of Latinx respondents indicating how returning to in-person school made it easier for them to access period products [25]. Such findings underscore the importance of new policy measures being introduced across numerous U.S. states that provide menstrual products to vulnerable populations in schools, prisons, and homeless shelters [37]. The mid-sized city of Ann Arbor, Michigan went even further with progressive action to address period poverty, passing a law in 2021 which mandates the provision of free menstrual products in all public bathrooms, including local businesses [38].

Varied progress has been found with respect to ensuring that government benefits can be used for the purchase of menstrual products. At present, only one U.S. state, Illinois, has passed legislation to ensure that both SNAP and WIC benefits can be used for the purchase of menstrual products [39]. This means that most low-income Americans are still unable to use governmental benefits to buy these items. Notably, in March 2020, with the passage of the Coronavirus Aid, Relief and Economic Security (CARES) Act, the U.S. government classified menstrual products as medical expenses, enabling the use of flexible spending accounts (FSA), health savings accounts (HSA), and health reimbursement arrangements (HRA) for the purchase of products [40]. While this indicates progress, only select people can benefit. For example, FSA and HRA accounts are employer-based and thus contingent on your employment status and all three require that you must have funds to buy products to take advantage of pre-tax programs.

Our findings also served to highlight how menstrual product stockouts and supply chain disruptions impacted women’s regular access to needed menstrual products. These insights regarding inadequate quantities of menstrual products and intermittent stockouts were also well-documented by the national media in the U.S. [23, 41, 42]. Not surprisingly, reports also suggested that many girls and women became more dependent on social service providers, such as food banks or non-profit organizations, for obtaining period products [42, 43]. For example, a non-profit named *I Support The Girls*, which operates in all 50 U.S. states, reported a 35% increase in requests in period products since the start of the pandemic [40]. Similar trends were documented by community-based non-profits operating in several U.S. cities, including Philadelphia, St. Louis, and Denver [44–46]. Little evidence is available, however, on the actual accessibility of menstrual products within the larger food bank systems operating across the U.S., including whether such actors are reliable resources for addressing unmet menstrual product needs. This includes examining how the pandemic may have hindered both the quantity of volunteers and menstrual product donations being made; resources that are integral for community-driven organizations such as food banks.

Most women in our study described having to adapt their menstrual management practices during the first year of the pandemic. For many women this included shifting the types of materials used to cheaper and poorer quality options and in some cases, makeshift materials. The emergence of stay-at-home policies or limitations in mobility resulted in many women spending more time within their homes than before the pandemic. In some cases, the reductions of movement may have made the dependence on makeshift or inferior menstrual products more manageable. However, as the pandemic progressed and people began to return to work, the coping strategies involving lower quality materials for managing monthly blood flow may have been less effective and more anxiety provoking. This was especially problematic if women had employment that was more physically active or required interfacing with individuals given fears of leaks. Our study also indicated that some women were receptive to trying reusable menstrual products, including menstrual cups and period underwear, for the first time during the first year of the pandemic. Despite varying acceptability, such findings may suggest a growing openness to more sustainable menstrual products, especially when framed as a cost-effective period management solution.

Our findings also found that both the stress of worrying about access to menstrual products and dependence on inferior or makeshift menstrual materials amplifies women’s experiences of shame or embarrassment. Some pre-pandemic research examining the relationship of unmet menstrual needs and mental health further validates these findings. A national survey of 471 college-attending women in the U.S. found that struggles with accessing period products were associated with heightened experiences of depression [47]. Such findings are comparable to the evidence available on the negative mental health impacts associated with food insecurity [48]; an issue compounded by the COVID-19 pandemic[49–51]. Qualitative research conducted with women experiencing homelessness in New York City also highlighted the social and mental well-being difficulties faced by women experiencing menstrual product insecurity. This includes how poor access to menstrual products, toilets, and laundering facilities may worsen one’s ability to “pass” as someone who is not homeless and thus engage in many daily living activities [52, 53]. Difficulties accessing menstrual products has also been found to lead many women experiencing homelessness to resort to stealing, panhandling, or using makeshift materials [53–55].

This study highlights how experiences of menstrual product insecurity may exacerbate menstrual stigma. Evidence suggests that societal discourse on menstruation can negatively impact an adolescent girl’s experience of menarche, including perpetuating the use of distancing language, such as euphemisms, when discussing menstruation, or a fixation on concealing one’s menstruating body [54]. A national survey of 165 women aged 18–37 years of age further describes this desire for secrecy, with respondents recalling experiences of being socialized into “self-silence” about their periods when growing up, a behaviour which many carried on into adulthood [11]. This desire for discretion became more challenging to maintain upon experiencing menstrual product insecurity during the first year of COVID-19. A lack of consistent access to quality menstrual products put many women, some for the first time in their lives, at risk for disclosing their menstrual status to others. Financial challenges also likely resulted in some women having to initiate direct conversations with their partners on needed household expenditures, which includes menstrual products; conversations which may not have occurred in pre-pandemic times. Previous research has also found that many people are shy or uncomfortable when asking for menstrual products from food banks due to personal embarrassment that they cannot afford their own and ongoing period stigma [55].

## Limitations

There are several limitations to note. First, there is potential for selection bias amongst the participants of this qualitative study. Given that this sample was self-selected, it is possible that the respondents included a higher proportion of individuals that were comfortable or willing to discuss a sensitive topic like menstruation and period poverty. Second, given the high levels of stress and the unprecedented living experiences caused by the COVID-19 pandemic, many respondents may have experienced recall bias. This includes issues with being able to accurately remember when and how specific events may have occurred and impacted their daily lives and perceived well-being. Third, as the study participants were cisgender women, our findings cannot provide insights on the experiences of trans and gender nonbinary menstruating individuals, and potentially the heightened, menstruation challenges they may face. Fourth, given that the study participants were required to be 18 years of age or older (an ethical requirement of the larger CHASING COVID study), we were unable to document learning on the experiences of adolescents that menstruate; a demographic with unique menstrual experiences both within their households and school settings. Lastly, all study participants were English speakers, literate and had some amount of digital accessibility (e.g., email and/or cellphones), thus highlighting that this sample is not representative of many low-income or non-English speaking populations who menstruate. Additional research should be conducted which aims to include these often-vulnerable menstruating populations.

## Conclusion

This qualitative study provided insights on women’s experiences with menstrual product insecurity during COVID-19. Three key recommendations for research, practice and policy include: One, expanded research on the impact of menstrual product insecurity on the lives of people who menstruate, including its prevalence and how it impacts their well-being, education, and employment experiences; Two, a re-framing of menstrual products as “essential items” that is incorporated into increased menstrual product access policies and safety net resources, including state and city-level legislation and the prioritization of these items by social service organizations; Three, improved national attention towards addressing menstrual stigma through education in schools and media advocacy campaigns. Addressing menstrual product insecurity is a crucial step towards ensuring that all people that menstruate can attain their most basic menstrual health needs.

## Data Availability

The dataset generated during this assessment are not publicly available due to the highly personal nature and detailed description of the very personal experiences in relation to menstruation which formed the basis of the qualitative interview and focus group guides. Furthermore, during the informed consent process, participants did not consent to making the data publicly available. Nonetheless, de-identified data may be made available by the corresponding author on reasonable request.

## Abbreviations

CARES: Coronavirus Aid, Relief and Economic Security
FSA: Flexible spending accounts
HRA: Health reimbursement arrangements
HSA: Health savings accounts
IDI: In-depth Interview
SNAP: Supplemental Nutrition Assistance Program
SEM: Socio-ecological model
U.S.: United States of America
WIC: Women, Infants, and Children
